# Role of the “High Institute of Public Health” during the COVID-19 Pandemic: A Case from Egypt

**DOI:** 10.5334/aogh.4387

**Published:** 2024-05-24

**Authors:** Engy Mohamed El-Ghitany, Eman A. Omran, Noha Ahmed EL Dabbah, Samar M. Aborhyem, Nashwa Fawzy Abd El-Moez Azzam

**Affiliations:** 1 Department of Tropical Health, High Institute of Public Health, Alexandria University, Alexandria, Egypt; 2 Department of Microbiology, High Institute of Public Health, Alexandria University, Alexandria, Egypt; 3 Department of Health Administration and Behavioral Sciences, High Institute of Public Health, Alexandria University, Alexandria, Egypt; 4 Department of Nutrition, High Institute of Public Health, Alexandria University, Egypt

**Keywords:** COVID-19, public health institute, research, postgraduate, community services

## Abstract

**Background:**

The High Institute of Public Health (HIPH), as a post-graduate academic institute, was affected by the COVID-19 pandemic in several aspects. This paper describes the effect of COVID-19 on the three main domains of HIPH: research, education, and community services. Documenting the activities and practices of the HIPH during the pandemic reflects the degree of resilience and preparedness against possible future global emergencies. Despite its importance for policymakers, such data is lacking from similar institutes in the Middle East, including Egypt.

**Methods:**

An extensive search in four popular scientific databases (Google Scholar, PubMed, Scopus, and Scival) was conducted to extract publications by authors affiliated with the HIPH using relevant keywords. Records were reviewed to collect data on the educational process as well as data on community services (convoys, campaigns, seminars, and workshops held by HIPH staff). All the mentioned activities were described, analyzed and compared before and during the pandemic to study the impact of the pandemic on the HIPH, as an example of a postgraduate institute.

**Results:**

The total numbers of COVID-19-related publications in Scopus by authors affiliated with the HIPH were 115 publications, the majority of which were research articles in the ‘Medicine’ and ‘Immunology and Microbiology’ domains. Most of them focused on assessing the relationship between the pandemic and quality of life, and prevention and treatment of COVID-19 (22.2% each). Publications on COVID-19 by HIPH researchers during the pandemic constituted 33.1% (115 publications) of the total publications by Alexandria University. Among the top ten authors on COVID-19 at Alexandria University, four were HIPH affiliated. The year 2022 witnessed the most frequent publications on COVID-19 by HIPH (51/115 publications, 44.3% of all COVID-19 publications by the HIPH on Scopus). All program courses were taught online during the year 2019–2020 (343 courses). HIPH provided several community services during the pandemic, which included 16 convoys in the poorer areas of Alexandria that served more than 1250 beneficiaries. Their goals were raising health awareness on COVID-19 vaccination, health education, and environmental assessment.

**Implications for Policy & Practice:**

This paper is the first of its kind by members of the High Institute of Public Health, Alexandria University. It provides baseline data for future similar work and is a documentation of the compilation of efforts during the COVID-19 pandemic that gives baseline data for public health assessment and planning by policy makers.

## Introduction

The COVID-19 pandemic, which swept the globe by the end of 2019, had a profound impact on public health systems as well as educational bodies worldwide. Although the pandemic was announced by the World Health Organization (WHO) to have ended in May 2023, its impact has had long-lasting effects [1].

The pandemic posed grave challenges but has ultimately also forced some institutions to reach resolutions and innovative strategies that did not exist before the pandemic. Most of the world universities have experienced at least one of the following phases: abrupt closure of the campus, opening the campus for specific tasks, and operating with social distancing measures [2].

According to a report by the International Association of Universities, 25% of teaching classes were suspended and 7% ultimately cancelled due to unpreparedness [3]. The infrastructure gap and the need for the learning experience were the main constraints that faced universal shift from face-to-face to online teaching. Poor learning outcomes caused frustration that required massive training programs to strengthen the use of digital tools by the educators to create virtual courses [2].

Administration and management staff at the universities continued their activities from home which required digitalization of the administrative processes. Universities diversified their financing resources to overcome lost income from conferences, sports activities, exhibitions, cafeterias, and parking [2,3].

In Egypt, the higher education sector includes 17 public as well as several national and private universities, which all offer distinguished academic degrees of master’s, doctorate, and higher diplomas for postgraduate students. Established in 1938, Alexandria University is the third oldest university in Egypt as well as being the second largest one, with a total of 143,553 undergraduate and 8,752 postgraduate students [4]. According to the Academic Ranking of World Universities (ARWU), also known as the Shanghai Ranking, in 2023, Alexandria University ranked 601–700 worldwide, with Public Health among its best ranked subjects (201–300 rank).^1^ Alexandria University encourages researchers through providing financial awards in case of publishing research in top-quartile journals as well as when receiving high citation rates.

The main governmental bodies that directly fund scientific research in Egypt are the Science, Technology and Innovation Funding Authority (STDF), the Academy of Scientific Research and Technology, and the Initiative for Cooperation between Companies and Research Institutions (ITAC) of the Information Technology Industry Development Authority. However, most researchers perform their studies without financial support from external bodies [5].

In 1963, the High Institute of Public Health (HIPH), affiliated with Alexandria University, became the first post-graduate institute offering a master’s degree in public health in the Eastern Mediterranean Region. Earlier, in 1956, the HIPH was founded by the Egyptian Ministry of Health to support the healthcare systems in Egypt and other countries in the region. The HIPH is unique with its diverse subspecialties that may not be available in any of the community health/medicine departments of faculties of medicine in Egypt [6]. According to a study published in 2021 which mapped the university-based Master of Public Health Programs in the Arab world, only two institutes in Egypt were reported to offer these programs: The High Institute of Public Health at Alexandria University and the Institute of Global Health and Human Ecology at American University in Cairo, established in 2018 [7].

The scope of HIPH encompasses education, research, and community service. Its academic role extends to national and regional levels, creating and transferring knowledge, skills, and expertise in public health through its multidisciplinary programs. The HIPH has dynamic partnerships with health and environmental sectors and organizations for the provision of comprehensive community health services. The institute includes 10 departments, which are: Epidemiology, Biostatistics, Tropical Health, Primary Health Care, Microbiology, Occupational Health and Industrial Medicine, Environmental Health, Nutrition, Health Administration and Behavioural Science, and Family Health. The HIPH provides diploma, master’s, and doctorate degrees in Public Health for physicians, while some of those programs are also offered to non-physicians. Those degrees are thesis-based and sometimes are part of research projects [6]. Research topics in the HIPH align with the United Nations 17 Sustainable Development Goals (SDG), as part of finding scientific solutions for global problems. This is an integral part of university goals worldwide and acts as one of the university ranking criteria. Research topics in the HIPH include, but are not limited to, those on communicable as well as non-communicable diseases (NCD), which according to the WHO report on Egypt’s health problems are estimated to account for 82% of all deaths in Egypt [8].

During the COVID-19 pandemic, the HIPH faced the challenge of the need for social distancing and lockdown, which necessitated the abrupt shift from face-to-face campus education to distant learning. Moreover, the domains of research and community service were directed purposefully toward COVID-19 research and containment.

After the pandemic was over, we conducted this present research paper to document the efforts and experience of the HIPH as a postgraduate research and public health institute in response to the COVID-19 pandemic. This paper was inspired by a practice paper published in *Annals of Global Health* in 2021 and was titled ‘Mapping University-Based Master of Public Health Programs in the Arab World’ [7]. We believe that our work continues the documentation process of the activities and role of public health educational institutes in the Middle East, especially during global public health emergencies such as COVID-19. Such documentation is largely lacking in the current literature. This paper aimed to navigate the impacts (whether positive or negative) of the pandemic on the institute’s research output, capacities, services, and activities. This would indicate the potential role and the preparedness of the institute in facing possible future public health emergencies.

## Methods

Several meetings were held by the researchers of this study to brainstorm and determine the elements required to be included in each part of the data collection sheet. The activities of the HIPH, in its three main domains; namely: Research, Education, and Community Services were retrospectively investigated and analysed to cover the period of the COVID-19 pandemic (end of 2019 until May 2023) as well as the total publications by HIPH researchers as far as online databases retrieved. The pattern of publication rate was compared before and during the pandemic, with emphasis on the main themes of publications during the pandemic.

### Research

Regarding the ‘Research’ domain, we searched scientific publications in four popular scientific databases including Google Scholar,^2^ PubMed,^3^ Scopus,^4^ and Scival,^5^ for relevant articles (all in English language; no other language is used for publishing by HIPH researchers) published by authors affiliated with the High Institute of Public Health. All search attempts were carried out on the same day (17 December 2023) for standardization and fair comparison.

For Google Scholar and PubMed, a search for the affiliation ‘High Institute of Public Health’ was done. Scopus database was curated for some organizational information on the HIPH, using the ‘Organization profile page’ to obtain information about the Institutional ID number. In addition, total publications as early as online articles appeared until 2023 were retrieved. Secondly, publications during the COVID-19 pandemic were grouped and its search date was set to be between 2020 and 2023. The third group of publications included studies done specifically on COVID-19. Search for this group was done using the following keywords: ‘COVID-19,’ ‘SARS-CoV-2,’ and ‘Coronavirus.’ This third group was sorted by two of our authors according to five categories: 1) Diagnostic and immunological studies, 2) vaccine-related studies, 3) epidemiological and survey studies, 4) effect of COVID-19 pandemic on the quality of life, and 5) prevention and treatment studies.

The number of studies related to the Sustainable Development Goals (SDGs) were also retrieved from Scopus, as well as the h-index of publications and their citation frequencies. Since the Scopus database is interactive and allows the analyses of the retrieved data to present them as graphs, Scopus-generated figures were added to our Results section.

Using the Scival database, the total publications on COVID-19 by all affiliations with Alexandria University were obtained. Their citation frequency and Field-Weighted Citation Impact were identified. The percentage of contribution by the HIPH was calculated by dividing the number of COVID-19 publications by HIPH members over the total COVID-19 publications by all Alexandria University affiliated faculties/institutes.

### Education

The numbers of students registered for diploma, master, and doctorate degrees over the past three years before the pandemic (2017–2019) and during the other three years of the pandemic (2020–2023) were retrieved from the records. Data on the educational process in the HIPH during the COVID-19 pandemic were collected from administrative departments responsible for student records and registration, the Quality Assurance Unit, the Office of Graduate Studies and Research, and the Measurement and Assessment Unit, HIPH.

### Community services

Data on community service during the pandemic were collected from the Office of Community Service and Environmental Development Affairs, HIPH. These data included the campaigns and convoys (their number, size of target population, and scope of action) as well as data on all sorts of educational material on COVID-19 provided to the public on social media, printed materials, and elsewhere. The number and scope of seminars and workshops held by the HIPH staff members (either at the HIPH premises or outside it) during the pandemic on COVID-19 were also included in this part of the data collection.

In line with the ongoing pandemic at the time, the 10th International Conference of the HIPH in 2022 was titled ‘Telehealth Novel Public Health Perspectives in the Third Millennium.’ Data on the number and nature of lectures related to COVID-19 presented during the conference sessions were also recorded in our data sheet, as well as the number of attendees.

Our research paper was not submitted for ethical clearance since it is limited to a review of published information and not a study of human subjects.

## Results

### Research

Databases were extensively searched for publications by the HIPH staff members. Search results in Google Scholar yielded a total of 6450 publications by the HIPH researchers (the oldest publication available on that database dates to 1952) while less results were retrieved by PubMed (total of 889 publications, the oldest of which dates to 1989).

Searching the Scopus database, the Affiliation ID for the Institute was found to be designated as ‘60004039,’ and its oldest publication dates to 1957. The total number of publications on Scopus with authors affiliated to the HIPH reached 1238, with a total of 512 authors. On analysis of HIPH publications about the SDG, Scopus showed that all of the 17 SDGs were covered by HIPH publications, with the highest frequency being published under the SDG ‘Good health and well-being,’ Goal 3 (492 documents), followed by the SDG ‘Partnership for the goals,’ Goal 17 (236 documents), and then the SDG ‘Clean water and sanitation,’ Goal 6 (88 documents).

**Figure 1:**
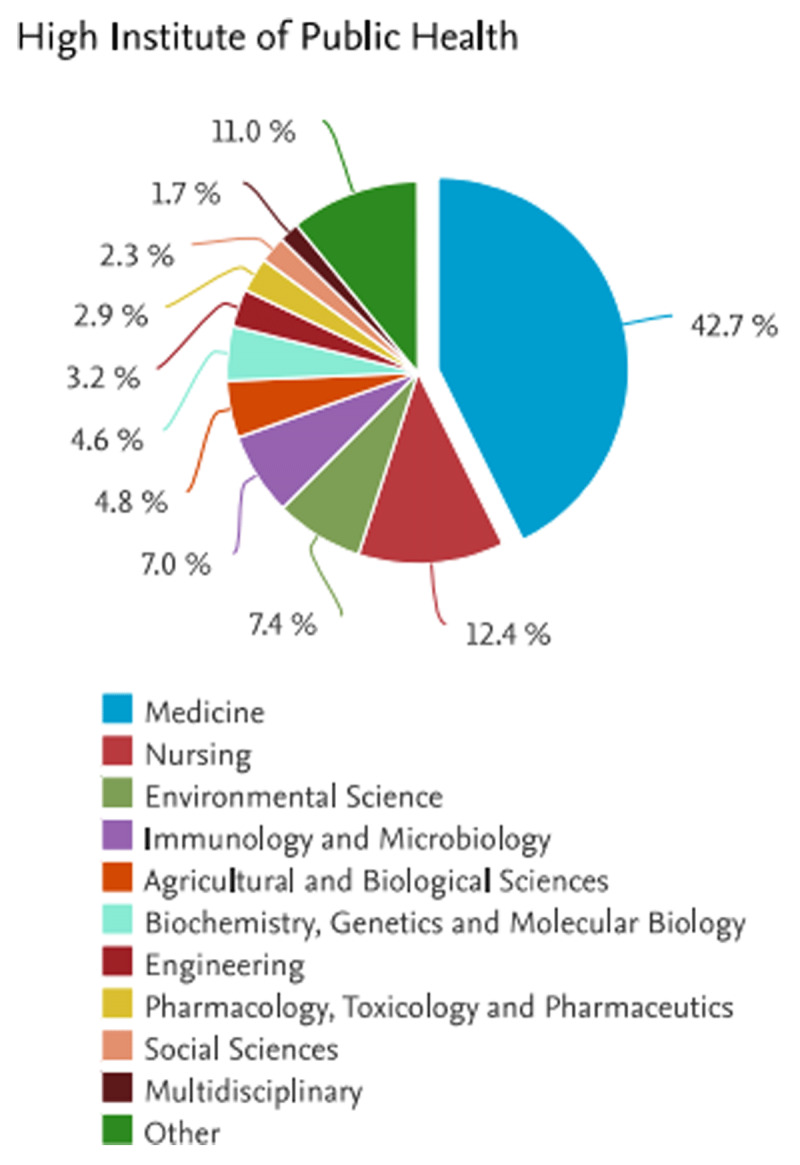
Total publications by the HIPH researchers (1957–2024) indexed in Scopus database (17 December 2023)^6^.

Most publications were in the scope of medicine (883 publications, 42.7%), nursing (256 publications, 12.4%), environmental sciences (154 publications, 11.0%), and others (Figure 1). Publications in Scopus constituted 87.0% research articles (n = 1077), 5.4% review papers (n = 67), 2.6% book chapters (n = 32), 2.3% conference papers (n = 29), and 1.0% letters (n = 12) and the remaining publications were errata, editorials, and notes.^6^

**Figure 2:**
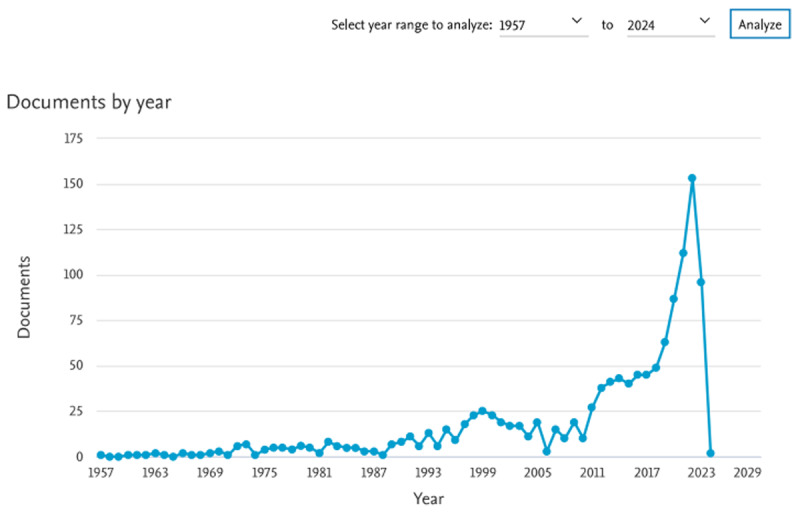
Total number of publications by the HIPH researchers throughout the years up to 2024. The frequency of annual publications spiked in 2022 with a total of 153 documents. (Scopus, 17 December, 2023)^6^.

The highest annual rate of scientific publications of all time was during the year 2022 (153 publications on Scopus) (Figure 2). In PubMed, 890 total publications were retrieved; the highest number (105) was during the year 2022. Of the 890, 107 articles were COVID-19 related.

**Figure 3:**
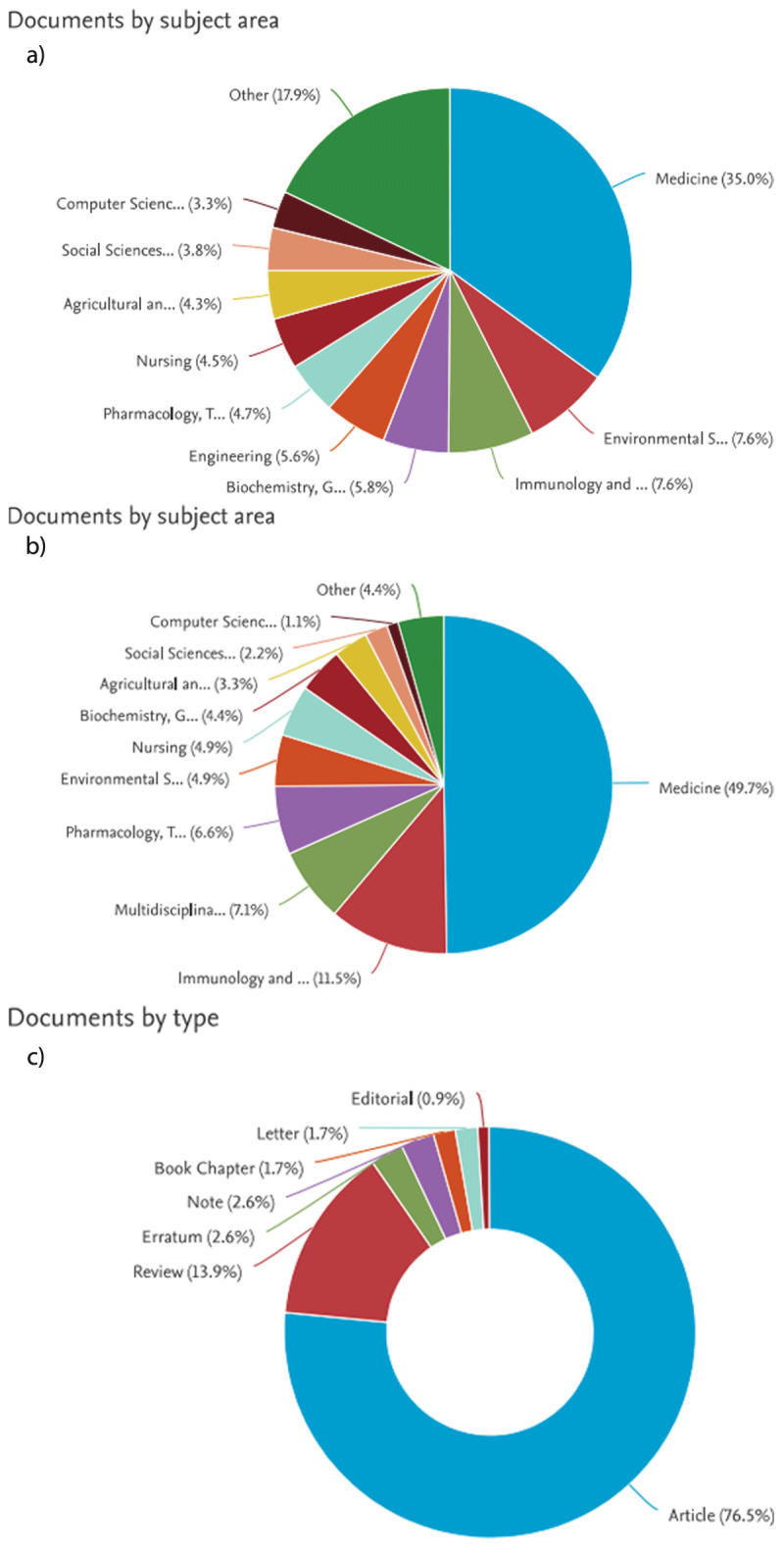
Scopus-generated figures **a)** Domains of publications by the HIPH researchers between the years 2020 and 2023 as in Scopus database (17 December 2023). **b)** Domains of publications by the HIPH researchers on the theme “COVID-19” (2020-2023) as indexed in Scopus database (on 17 December 2023). **c)** Types of publications by the HIPH researchers on the theme “COVID-19” (2020–2023) as indexed in the Scopus database (on 17 December 2023).

The total number of publications by the HIPH (on all topics) from 1 January 2020 until 17 December 2023 on Scopus was 450. The main domains of publications in order of frequency were: medicine (n = 277, 35.0%), immunology and microbiology (n = 60, 7.6%), environmental sciences (n = 60, 7.6%), and others (Figure 3a). Most publications comprised research articles (n = 348, 77.8%) followed by review articles (n = 42, 9.3%), book chapters (n = 29, 6.4%), and conference papers (n = 13, 2.9%). The h-index of those 450 publications was 28, according to Scopus and those documents were cited by 3640 research articles.^7^

**Figure 4:**
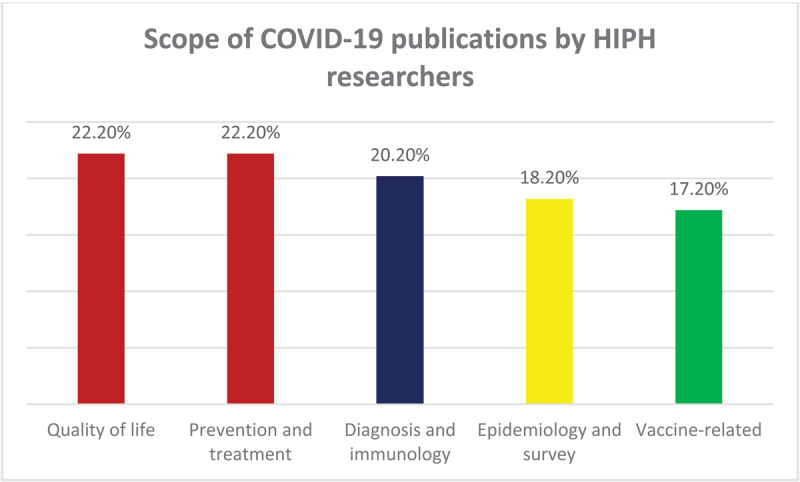
Scope of Scopus publications on COVID-19 by the HIPH researchers until 17 December 2023.

Most published online studies on COVID-19 by HIPH researchers were not theses-based (n = 115 publications on COVID-19 in Scopus, and 107 publications retrieved by PubMed) probably due to the need for more rapid publication at that time. Based on data extracted from Scopus, publications on COVID-19 (n = 115), represented 25.6% of all publications by the HIPH between 2020 and 2023 (n = 450) and from PubMed, it was calculated as 31.8% (107/337). The year 2022 witnessed the most frequent publications on COVID-19 by HIPH (51/115 publications, 44.3% of all COVID-19 publications by the HIPH on Scopus) (Figure 4). Out of the 115 publications, only three studies were randomized controlled trials, four were meta-analyses, six were systematic reviews, 13 were reviews and the rest were original articles and book chapters.^8^

Of those 115 COVID-19 publications in Scopus, 91 (49.7%) were in medicine, 21 (11.5%) in immunology and microbiology, 12 (6.6%) were in pharmacology, toxicology and pharmaceutics, and the rest belonged to other specialties (Figure 3b). Most of these publications comprised research articles (n = 88, 76.5%) and review articles (n = 16, 13.9%) (Figure 3c).

Seven publications were extracted from a project in 2020 on COVID-19. This project was funded by the Science and Technology Development Fund (STDF) (Project no. 43834) (2020), and its duration was 12 months and was titled: ‘An Exploratory Survey of COVID-19 Humoral Immunity among Egyptian General Population and Healthcare Workers.’ One of those project-derived articles was published on the Serotracker website, which is a WHO dashboard and data platform for SARS-CoV-2 serosurveys. That article was referred to by the WHO Serotracker as a national study from Egypt on COVID-19 seroprevalence.^9^ This project also contributed to capacity building since it funded the purchase of new laboratory equipment (enzyme-linked immunosorbent assay (ELISA) reader and washer) to perform immunological tests against SARS-CoV-2.

Another project on COVID-19 funded by Pfizer was carried out by HIPH researchers and was entitled ‘Epidemiological Survey and Genotypic of COVID-19 among Children in Alexandria.’

The majority of the 115 publications on COVID-19 focused on assessing the relationship between the pandemic and quality of life (23 publications, 22.2%), prevention and treatment of COVID-19 (23 publications, 22.2%), followed by diagnostic and immunological studies (21 publications, 20.2%), epidemiological and survey studies (19 publications, 18.2%), and vaccine-related studies (19 publications, 17.2%) (Figure 4).

Seven (6.1%) publications were on healthcare workers, and 11 (9.6%) were on hospitalized patients. Out of all 115 publications, one was a letter to the editor, and one was a qualitative study.

**Figure 5:**
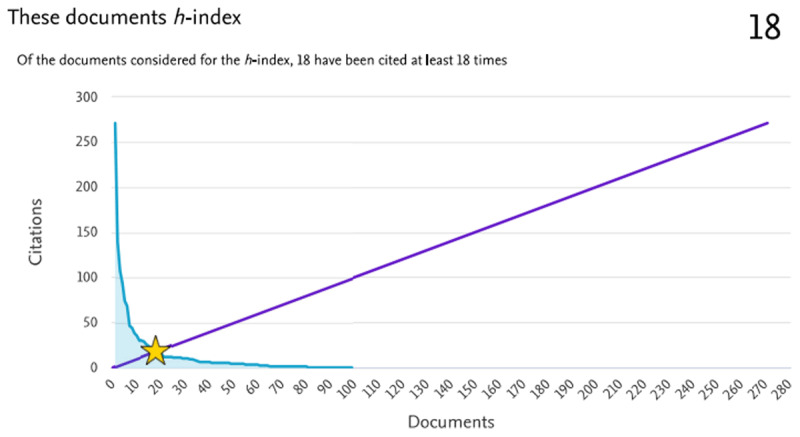
The h-index of the 115 publications by the HIPH researchers during the COVID-19 pandemic (2020–2023) indexed in the Scopus database (until 17 December 2023). The h-index equals 18 according to the figure.

Total citations on Scopus for all COVID-19-related publications by the HIPH staff reached 1565. The overall h-index for those 115 documents, according to Scopus, was found to be 18, meaning that 18 documents have been cited at least 18 times^10^ (Figure 5).

It is noteworthy to mention that, searching the Scival database for the total publications on COVID-19 by all affiliations of Alexandria University during 2020–2023, the total number of publications was 347 with a total citation count of 6404 and a Field-Weighted Citation Impact of 2.86.^11^ This indicates that publications on COVID-19 by HIPH researchers during that same period constituted 33.1% (115 publications) of the total publications by Alexandria University. Among the top ten authors on COVID-19 at Alexandria University, four were HIPH affiliated.

### Education

The Office of Postgraduate Studies and Research was responsible for planning, implementing, and monitoring a comprehensive online teaching strategy during the pandemic. The Measurement and Assessment Unit organized several training workshops to facilitate the conversion of all taught courses to virtual mode. Multiple tutorials were held to train the academic staff on how to teach through the available learning platforms: Microsoft Teams and Google Classroom. The staff members were trained on how to prepare their courses, schedule online meetings, and share all needed learning materials and assignments with the students in addition to training them on how to create, administer, and mark online electronic exams to reduce the potential risks of infection.

Starting from the spring semester of 2019–2020, around 80% of all the institute courses taught during spring were taught online through the different available learning platforms such as Microsoft Teams, Google Classrooms, Zoom, and eLearning Alexu, with a total number of 60 courses. Later, during the academic year 2020–2021, 100% of all the institute courses were taught online with a total number of 343 courses. In the following academic years, 2021–2022 and 2022–2023, the HIPH followed the hybrid learning strategy, with 35% face-to-face learning and 65% online learning.

All faculties and institutes affiliated with Alexandria University (including the HIPH) followed standard precautionary measures during the exams for the academic year 2019–2020. These included several parameters, such as temperature recording, social distancing, proper ventilation, disinfection, and provision of alcohol dispensers, health education (through WhatsApp groups, the official website of the institute, and posters), and the use of an isolation room for suspected cases who were later referred to the central COVID-19 clinic defined by Alexandria University after the provision of adequate first-aid medications. A medical committee was also formed from specialist doctors at the institute to follow up on the health condition of students during the exams. Exams were postponed for students who were suspected of having COVID-19. Older staff members (65 years or older), immunocompromised ones, and those with active COVID-19 infection were all exempted from any work related to exams.

### Community Services

Among other faculties and institutes of Alexandria University, the HIPH participated in the development of a comprehensive plan to control the COVID-19 pandemic on all university premises. A team of specialized professors from different departments participated in constructing the University’s strategy to control COVID-19.

Moreover, a team of professors from several departments at the HIPH designed a questionnaire for ‘Epidemiological Surveillance and Clinical Follow-Up of COVID-19 Cases in Alexandria University Hospitals.’ The questionnaire was disseminated among all the employees in the HIPH, and data was extracted to identify suspected cases that were later followed up by weekly telephone calls with advice being given on appropriate diet choices, hygiene practices, and personal disinfection measures.

During the period 2020 and 2022, six seminars were held at the HIPH that focused on the situation analysis of COVID-19, infection preventive measures and treatment modalities, impact of COVID-19 on non-communicable diseases, dynamic changes in COVID surveillance, and the new viral variants. Some staff members also participated in activities outside the premises of the HIPH to educate the public on COVID-19, and these included three seminars and appearing as guests in three television and radio shows.

Moreover, a video recording as well as a booklet were composed by staff members of the HIPH with educational material on the modes of transmission, clinical presentation, and preventive measures of COVID-19 in collaboration with the faculty of fine arts. A Facebook page^12^ was created and moderated by HIPH staff members who posted regularly on the page educational materials related to COVID-19 as well as other interesting healthy advice. It also conducted a questionnaire regarding COVID-19 vaccination coverage and enhancers and barriers to vaccination. The page was created in Arabic to engage non-English-speaking people in the community and have better public accessibility. It had a total of 2865 active members.

The HIPH 10th International Conference in 2022 had six of its oral presentations revolving around the COVID-19 theme. The conference was attended by around 500 public health and healthcare workers, and it tackled topics on diagnostic means of COVID-19 as well as real-life experiences of healthcare workers in hospitals and laboratories during the pandemic. Additionally, the conference theme was inspired by the dictated digital transformation because of the COVID-19 pandemic.

The HIPH was also represented in October 2022 at the workshop ‘Egypt’s National Health Research Prioritization,’ which was held by the Ministry of Health and Population (Training and Research Sector) and during which COVID-19 was included as one of the top national health priorities.

As an integral part of the activities of the Community Service and Environmental Development Affairs, HIPH, convoys and campaigns took place during the pandemic to help individuals in poorer areas of Alexandria. Sixteen convoys were organized and delivered by the HIPH and have served more than 1250 beneficiaries. The convoys helped residents to register on the electronic national database for vaccination against COVID-19. In addition, data was collected regarding demographic, social, health, and environmental conditions as well as reasons for vaccine hesitancy. Health education sessions were held in various specialties and medical examination was provided for residents along with screening for co-morbidities such as hypertension, diabetes, obesity, growth and developmental delay, parasitic infestations, and the like. Assessment of indicators of air pollution, noise, and water quality was carried out as part of the environmental evaluation of the residential areas. Reports on the health and environmental survey analyses were shared with local health and other relevant authorities for appropriate health and environmental enhancements in those areas.

## Discussion

The COVID-19 pandemic has triggered higher education systems across different parts of the globe to adapt through online education. In our study, online education might have partially contributed to the noticeably increased number of registered students at the HIPH during the academic year 2021–2022 compared to its previous years (Figure S1 a–c). Fear of COVID-19 and unstable conditions might have refrained students at the beginning of the pandemic from registering, although the educational process was online. During the second year of the pandemic (2021–2022), however, registration numbers increased despite the lower portion of online education. This might be due to the relative stability of people’s adaptation to the pandemic and their less fear, as well as the growing national and global importance of public health. We can conclude then that online education might not have been the sole factor affecting students’ desire to pursue postgraduate public health studies. Mok et al. reported a significant difference in proportion between information technology (IT) literacy and satisfaction with online learning experience [9], indicating the need for better technology infrastructure as well as training of users for more efficient online education.

Globally, students’ lives were furthermore affected negatively both mentally and socially through disruption of their interaction at universities [10]. One of the worrying concerns about online learning is whether this method is effective, specifically when compared to face-to-face classes [11]. One of the limitations of our study would be the fact that we did not assess the level of satisfaction of our students as well as their learning outcome parameters during the pandemic and compare these values to the conventional face-to-face education. This would have given a clearer view of the quality of online education. In a study from Hong Kong during the pandemic, by analysing the survey responses from 1,227 university students, most of the respondents felt dissatisfied with their online learning experiences and their effectiveness [9].

During the COVID-19 pandemic, a rise in scientific publications was noted worldwide and was similarly noted in HIPH research output, as the spike of scientific publications of all time was during the year 2022 (153 publications on Scopus and 105). This might have been because several institutions worked online and routine work was interrupted, diverting efforts to scientific research. Globally, studies on COVID-19 were rigorously published during the pandemic as this was the hot topic at that time. This pattern was also noted in the case of publications by the HIPH. In our study, most published online studies on COVID-19 by HIPH researchers were not theses-based (n = 115 publications on COVID-19 in Scopus, and 107 publications retrieved by PubMed) probably due to the need for more rapid publication at that time. Publications on COVID-19 by HIPH researchers on COVID-19 constituted 33.1% (115 publications) of the total publications by Alexandria University on the same topic. Among the top ten authors on COVID-19 at Alexandria University, four were HIPH affiliated which underscores the high research calibre of HIPH staff.

Similar to our findings, in their meta-analysis, Raynaud et al. reported a dramatic increase in the number of publications from February to April 2020 from a weekly median number of publications of 4.0 (IQR: 2.8–5.5) to 19.5 (IQR: 15.8–24.8) (p < 0.001), and reported that the pandemic was associated with a 18% decrease in the production of non-COVID-19 research. They also reported a decrease in the number of original articles [12].

In our institute, vigorous research on SARS-CoV-2 diagnosis and immune response had a positive impact on improving the laboratory skills of researchers who participated in such studies. All community-based activities in the HIPH were also devoted to COVID-19 issues. Health education on prevention of the infection was a major target for staff members.

## Conclusion

To sum up, the COVID-19 pandemic opened new avenues of educational and research experiences for HIPH staff members and students as well as the reinforcement of the HIPH’s role in community service and public health education. This study, not only documents what has been done by the HIPH during the pandemic, but also highlights the implications and needs for future development of public health education and research. As for the HIPH researchers, whether this pattern of high publication rates (as in 2022) will remain or decline is not easy to predict. It requires a few more years to compare publication rates and determine if this rise was only temporary.

## Additional File

The additional file for this article can be found as follows:

10.5334/aogh.4387.s1 Figure S1. Annual numbers of registered students at the HIPH before and during the COVID-19 pandemic.

## Footnotes

https://www.shanghairanking.com/institution/alexandria-university.

https://scholar.google.com/intl/en/scholar/about.html.

https://pubmed.ncbi.nlm.nih.gov/?term=high+institute+of+public+health++egypt&sort=relevance.

https://www.scopus.com/affil/profile.uri?afid=60004039&offset=1&sid=9e5d743df6c8751dae4f7e38ba0cc8f3&origin=AffiliationNamesList&txGid=79de3b0826c6b43cd527806bb308c219.

https://www.elsevier.com/products/scival.

https://www.scopus.com/term/analyzer.uri?sid=69c3618657171be471c2445f225ce39a&origin=resultslist&src=s&s=AF-ID%2860004039%29&sort=plf-f&sdt=a&sot=aff&sl=15&count=1238&analyzeResults=Analyze+results&txGid=bd3fec1fd3053af84e7b858b497fd050.

https://www.scopus.com/term/analyzer.uri?sid=eef90bd6def2fd8a4d767a8ea71e7b8f&origin=resultslist&src=s&s=AF-ID%2860004039%29&sort=plf-f&sdt=cl&sot=aff&sl=15&count=456&analyzeResults=Analyze+results&cluster=scopubyr%2c%222023%22%2ct%2c%222022%22%2ct%2c%222021%22%2ct%2c%222020%22%2ct&txGid=5357ae841de94727973e2d23ef64a3aa.

https://www.scopus.com/results/results.uri?sort=plf-f&src=s&st1=high+institute+of+public+health&sid=85f4c535b3d3e7fee09bc9d2f4b98a56&sot=b&sdt=cl&sl=41&s=%28AFFIL%28high+AND+institute+AND+of+AND+public+AND+health%29+AND+TITLE-ABS-KEY%28covid+OR+SARS+OR+corona%29%29&origin=resultslist&editSaveSearch=&yearFrom=2020&yearTo=2023&sessionSearchId=85f4c535b3d3e7fee09bc9d2f4b98a56&limit=10.

https://serotracker.com/en/unity (Accessed on 17 December 2023).

https://www.scopus.com/pages/citationOverview?key=0989f22b-3ed8-4d16-952c-562cd8c6ed41&origin=resultslist.

https://www.scival.com/overview/topics?uri=Institution/104003.

https://www.facebook.com/med.inf.144?mibextid=LQQJ4d.
