# Supplementary material for: Role of the “High Institute of Public Health” during the COVID-19 Pandemic: A Case from Egypt
Source: Ann Glob Health. 2024 May 24;90(1):33. doi: 10.5334/aogh.4387 (PMC11122695; doi:10.5334/aogh.4387)
Supplement: Figure S1. — Annual numbers of registered students at the HIPH before and during the COVID-19 pandemic. [file agh-90-1-4387-s1.pdf]

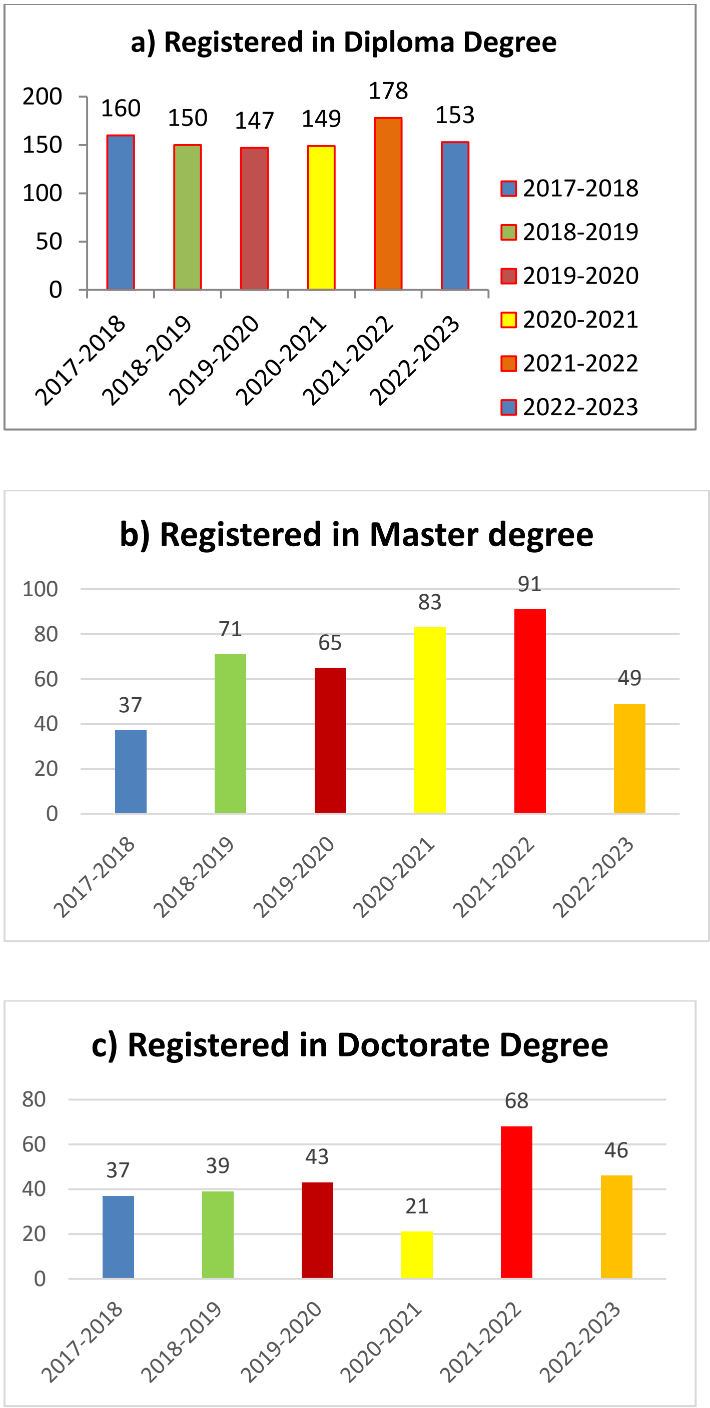

Figure (S1): Annual numbers of registered students at the HIPH before and during the COVID-19 pandemic
